# Application of Paper-Supported Printed Gold Electrodes for Impedimetric Immunosensor Development

**DOI:** 10.3390/bios3010001

**Published:** 2012-12-27

**Authors:** Petri Ihalainen, Himadri Majumdar, Tapani Viitala, Björn Törngren, Tuomas Närjeoja, Anni Määttänen, Jawad Sarfraz, Harri Härmä, Marjo Yliperttula, Ronald Österbacka, Jouko Peltonen

**Affiliations:** 1 Center of Excellence for Functional Materials and Laboratory of Physical Chemistry, Department of Natural Sciences, Åbo Akademi University, Turku, Finland; E-Mails: bjorn.torngren@abo.fi (B.T.); anni.maattanen@abo.fi (A.M.); jawad.sarfraz@abo.fi (J.S.); jouko.peltonen@abo.fi (J.P.); 2 Center of Excellence for Functional Materials and Physics, Department of Natural Sciences, Åbo Akademi University, Turku, Finland; E-Mail: ronald.osterbacka@abo.fi; 3 Division of Biopharmaceutics and Pharmacokinetics, Faculty of Pharmacy, University of Helsinki, Helsinki, Finland; E-Mails: tapani.viitala@helsinki.fi (T.V.); ylipertt@mappi.helsinki.fi (M.Y.); 4 Laboratory of Biophysics, Institute of Biomedicine and Medicity Research Laboratories, University of Turku, Turku, Finland; E-Mails: tuonar@utu.fi (T.N.); harri.harma@utu.fi (H.H.)

**Keywords:** paper electronics, nanoparticles, inkjet printing, immunoassays, impedance spectroscopy

## Abstract

In this article, we report on the formation and mode-of-operation of an affinity biosensor, where alternate layers of biotin/streptavidin/biotinylated-CRP-antigen/anti-CRP antibody are grown on printed gold electrodes on disposable paper-substrates. We have successfully demonstrated and detected the formation of consecutive layers of supra-molecular protein assembly using an electrical (impedimetric) technique. The formation process is also supplemented and verified using conventional surface plasmon resonance (SPR) measurements and surface sensitive characterization techniques, such as X-ray photoelectron spectroscopy (XPS) and atomic force microscopy (AFM). The article provides a possible biosensor development scheme, where—(1) fabrication of paper substrate (2) synthesis of gold nanoparticle inks (3) inkjet printing of gold electrodes on paper (4) formation of the biorecognition layers on the gold electrodes and (5) electrical (impedimetric) analysis of growth—all are coupled together to form a test-structure for a recyclable and inexpensive point-of-care diagnostic platform.

## 1. Introduction

In the field of clinical diagnostics, much focus is currently put towards the development of high performance biosensors that could provide low-cost and easy to use analytical tools for rapid, reliable and sensitive diagnosis of the clinically relevant analytes [1]. Generally speaking, biosensors can be classified as either enzymatic or affinity biosensors. In the former, enzymes are used as biorecognition element, whereas the latter are based on the affinity reaction resulting in the formation of a complex. Affinity biosensors include, e.g., immunosensors (antibody-antigen), DNA sensors and whole cell biosensors. The label-free affinity-based probing concepts for monitoring of antibody-antigen interactions are a subject of much academic and industrial level research, and they offer a potential alternative to the well-established and widely used enzyme-linked immunosorbent assay (ELISA). 

C-reactive protein (CRP) is a common marker of inflammation. Measuring and charting CRP values can prove useful in determining disease progress or the effectiveness of treatments. In addition, the acute phase proteins, such as CRP, display important biological functions with implications for the etiopathogenesis of many autoimmune diseases [2]. The level of autoantibodies directed against native or structurally altered forms of acute phase proteins have been shown to correlate with disease activity. For example, anti-CRP antibodies levels have been shown to be helpful to assess disease activity in systemic lupus erythematosus. 

Surface plasmon resonance (SPR) is a standard optical technique that allows for real-time monitoring of changes in the refractive index of a thin film close to a surface. This technique is used for the measurement of real-time, label free biomolecular (affinity) interactions. While one of the reaction partners is immobilized to the sensor surface, the other is passed over the immobilized layer as a solution. Bindings are measured as changes in the refractive index of the surface. Such detections are also possible by using electrical techniques, namely measurement of variation of the dielectric property of the two binding layers. This method is, in general, called impedimetric detection. The operations, development and applications of impedimetric immunosensors have been extensively reviewed in the literature [3,4,5,6,7,8]. Immunosensors based on impedimetric detection are considered as potential candidates because they possess attractive characteristics, such as cost-effective instrumentation, ease of miniaturization and integration into multi-array concepts. 

Paper-electronics or the science of creating electronics on paper by using various printing methods has gained in popularity over the last decade [9]. This opens up the possibility of building electronic platforms for cheap, disposable, recyclable applications. Biosensing, or medical diagnostics, is one such area where paper electronics can be of immense potential. There have been several reports worldwide on this topic. Screen-printing has been the most widely applied technique for producing paper-supported devices for, e.g., electrochemical glucose detection or electrochemical immunoassay [10,11,12]. It was recently shown by Määttänen *et al*. that inkjet printing can be utilized for the fabrication of a low-cost three-electrode platform on a recyclable paper substrate. It was also demonstrated that by modifying the printed gold working electrode, the platform could be used for various electrochemical analyses, e.g., for the detection of glucose [13].

In this article, we report on the formation and mode-of-operation of an affinity biosensor, where alternate layers of biotin/streptavidin/biotinylated-CRP-antigen/anti-CRP antibody are grown on printed gold electrodes on disposable paper-substrates. The response of the sensors is recorded using impedance analysis. The formation process of supramolecular protein assembly measured using impedance spectroscopy is furthermore compared and verified using surface plasmon resonance (SPR) measurements and surface sensitive characterization techniques, such as X-ray photoelectron spectroscopy (XPS) and atomic force microscopy (AFM). The article provides a possible biosensor development scheme where—(1) fabrication of paper substrate (2) synthesis of gold nanoparticle inks (3) inkjet printing of gold electrodes on paper (4) formation of the biorecognition layers on the gold electrodes and (5) electrical (impedimetric) analysis of growth—all are coupled together to build a test-structure for a recyclable and inexpensive point-of-care diagnostic platform.

## 2. Experimental Section

### 2.1. Print Substrate

A multi-layer coated paper developed for printed electronics was used as print substrate [14,15]. The paper contains components normally used in industrial paper-making. The paper was coated with materials that are commonly used in paper coatings, *i.e.*, mainly latexes and mineral pigments. In its core nature, the substrate used here is a natural fiber-based substrate, e.g., these papers are recyclable in a similar way as normal paper prepared for the conventional graphic industry. The most important physicochemical characteristics and examples of numerous applications in the field of printed electronics are described in previously publications [13,14,15,16,17,18,19,20]. In short, the main components in the nanoporous top coating (thickness ~3 µm) are kaolin pigment and styrene-butadiene latex binder. The total thickness and grammage of the paper substrate was about 130 µm and 126 g/m^2^, respectively. 

### 2.2. Synthesis and Characterization of the Gold Nanoparticles

All chemicals used for gold nanoparticle (AuNP) synthesis were purchased from Sigma-Aldrich and used without further purification. Dodecanethiol-stabilized AuNPs were synthesized following the procedure reported by Hostetler *et al*. [21]. Briefly, 1.4 mmol gold (III) chloride hydrate (HAuCl_4_·H_2_O) in 20 mL deionized water was added under vigorous stirring to 32 mL toluene containing 3.5 mmol tetraoctylammonium bromide (TOAB). The gold salt transferred into the organic phase, turning it orange brown. The water phase was discarded, 0.35 mmol dodecanethiol was added to the organic phase, and the mixture was stirred for 10 min. Sodium borohydride (NaBH_4_), 14 mmol in 20 mL water, was added to the mixture under vigorous stirring. The dark solution was thereafter stirred for 3 h, after which the organic phase was collected and evaporated. The remaining dark residue was redispersed in ethanol and filtered. The powder was washed with ethanol and acetone and dried in vacuum overnight.

The size and shape of the synthesized gold nanoparticles were characterized using a FEI Tecnai 12 transmission electron microscope (TEM). The TEM samples were prepared by dispersing the solid powder in xylene and applying a droplet onto a Cu mesh TEM grid. Image analysis of over 1,000 particles was carried out with the ImageJ software, giving a size distribution of 6.6 ± 2.3 nm. 

### 2.3. Inkjet Printing of Gold Electrodes

**Figure S1:**
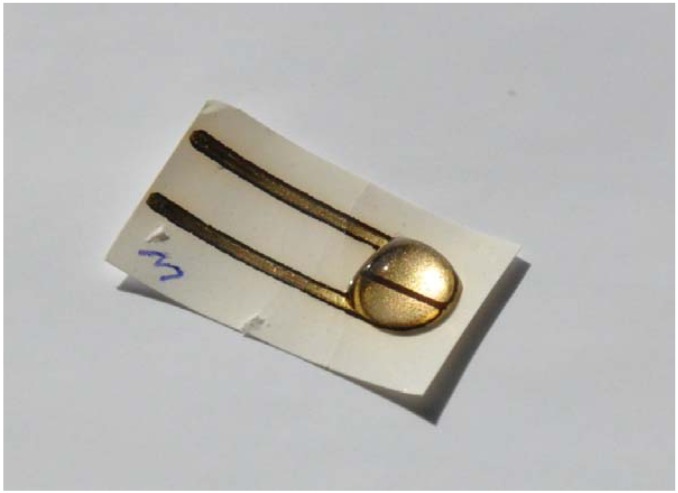
A photograph showing a printed gold electrode on a paper substrate. Also shown is a drop (40 µL) of buffer deposited over the electrode demonstrating how the immobilization of proteins and impedance measurement were conducted. The drop is confined over the target spot by a translucent hydrophobic PDMS layer around the perimeter of the electrode.

The AuNP ink for inkjet printing was prepared by the dispersing nanoparticles (15 wt%) in xylene (Sigma-Aldrich). Inkjet printing of the AuNP ink was performed with a Dimatix Materials Printer (DMP-2800, FUJIFILM Dimatix, Inc., Santa Clara, CA, USA). The printing was done in ambient conditions using a single nozzle, 10 pL drop volume, 27 ± 3 V firing voltage and a custom waveform to ensure optimal droplet formation. Printing was performed using a drop spacing of 20 µm. Sintering of the printed gold electrodes was carried out using a short-wave IR drier (IRT systems, Hedson Technologies AB, Sweden) consisting of three 30 cm long 2 kW strip light bulbs with distance between the sample and the lamp being about 20 cm. The sintering time was 20 s. The volume resistivity of the printed gold structures was 1.6 × 10^−7^ Ωm. A more detailed description of the fabrication, characterization and surface modification of the inkjet-printed paper-supported gold electrodes is given in a previous communication [19]. In addition, a hydrophobic and translucent polydimethylsiloxane (PDMS)-based ink (Dehesive 920, Wacker chemicals) was applied around the perimeter of the electrodes to confine the aqueous sample solutions over the electrode area during the protein immobilization and impedance measurements. The PDMS ink can be applied on a paper substrate by manual spreading or by using roll-to-roll compatible techniques, such as flexographic or inkjet printing [22]. A photograph of a paper-supported printed electrode used in this study is shown in the Supplementary Figure S1. Material cost for manufacturing the electrodes are ~4 cents/cm^2^ due to the low material consumption of inkjet printing, making them a really low-cost alternative to conventionally prepared electrodes.

### 2.4. Monothiols and Proteins

11-Mercapto-1-undecanol (MuOH, HS(CH_2_)_11_OH) was purchased from Sigma-Aldrich. Biotinylated hexa(ethylene glycol) undecane thiol (Biotin-PEG-thiol, HS(CH_2_)_11_(OCH_2_CH_2_)_6_NHBiotin) was obtained from nanoScience Instruments (Phoenix, AZ, USA). The thiols were used as received. Streptavidin was purchased from BioSPA (SPA Società Prodotti Antibiotici S.p.A., Italy). Anti-CRP antibody was received from Orion Diagnostica Ltd., Finland. CRP antigen stock 2.95 mg/mL in TSA-buffer (pH 8, 20 mM Tris 150 mM NaCl, 7 mM NaN_3_ and 0.2 mM CaCl_2_) was acquired from Orion Diagnostica Ltd., Finland. Biotinylated-CRP antigen (bio-CRP antigen) was prepared as follows: CRP antigen was concentrated to 10 mg·mL^−1^ and the buffer was exchanged to 25 mM carbonate buffer (pH 8.2) in Nanosep 30K (Pall, USA) centrifugal device. 1 mg of CRP antigen was biotinylated with (+)-Biotin N-hydroxysuccinimide ester (Sigma-Aldrich, USA). Biotin-NHS was dissolved in DMSO to give a final concentration of 11 mg·mL^−1^ and 10 μL aliquot was combined with CRP antigen. The reaction was mixed and allowed to progress for 5 h at room temperature. The bio-CRP antigen was purified with NAP5-column (GE healthcare, USA), and the buffer was exchanged back to TSA. The purified concentration was assayed to be 599 mg·mL^−1^.

HEPES-EDTA aqueous solution (10 mM HEPES (Sigma), 150 mM NaCl (Fluka), 1 mM EDTA (Sigma), pH 7.4) was used as a buffer solution in all the protein experiments.

### 2.5. Formation of Supramolecular Protein Layers on Printed Gold Electrodes

**Figure 1:**
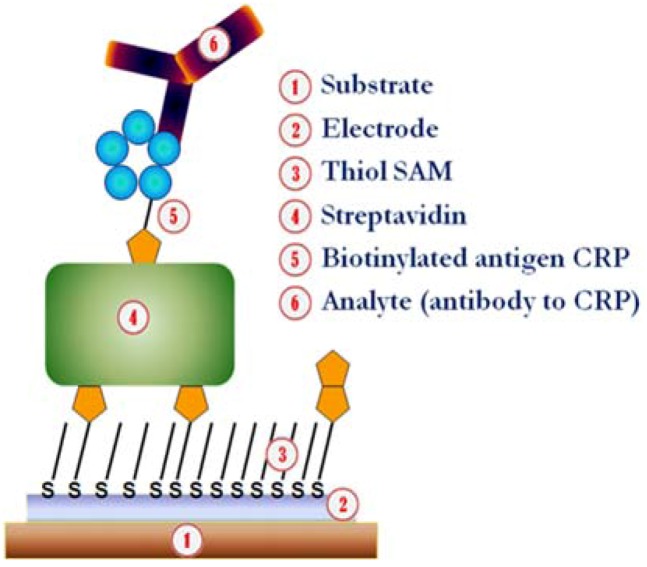
Schematic diagram of the structure (not to scale) of the supramolecular protein layers streptavidin, biotinylated c-reactive protein (CRP) antigen and the bound analyte (anti-CRP antibody) grown on biotinylated self-assembly monolayer (SAM)-covered printed gold electrodes on a paper substrate.

A schematic representation of the supramolecular protein layers is provided in Figure 1. The first layer consisted of an adsorbed biotinylated self-assembled monolayer (SAM) on the gold electrodes, formed from a MuOH:Biotin-PEG (85:15 mol%, 5 mM) (MBP) thiol solution in absolute ethanol (ETAX Aa, Altia). Before exposure to SAM solution, the gold electrodes were cleaned with plasma (air) flow (PDC-326, Harrick) for 2 min, rinsed with acetone and absolute ethanol and dried with nitrogen gas. The paper-supported electrodes were sealed between two silicon rings in a liquid cell with a cap to prevent evaporation, and the electrode surfaces were exposed to SAM solution (250 µL) for 16 h at room temperature in the dark. The MBP was adsorbed on the gold surfaces. After thiolation, the substrates were removed from the solution and rinsed immediately with absolute ethanol and dried with nitrogen gas. The MBP SAM was used as a starting point for the formation of supramolecular recognition assembly to ensure that the anchored streptavidin molecules remained biologically active and in their native conformations and formed a homogenous and strongly bound intermediate layer with a high binding capacity for subsequent immobilization of bio-CRP.

The second layer was formed by applying a 40 µL drop of streptavidin in HEPES-EDTA aqueous solution (2 µg/mL) over the electrode area. The immobilization of streptavidin onto the biotinylated SAM covered electrodes was conducted at +4 °C for 8 h. After immobilization, the electrode surface was rinsed with the HEPES-EDTA buffer solution and pure water (MilliQ) and dried with nitrogen gas.

The third layer was formed by applying a 40 µL drop of bio-CRP antigen in HEPES-EDTA aqueous solution (20 µg/mL) over the electrode area. The immobilization of bio-CRP antigen onto the MBP thiol/streptavidin layer covered electrodes was conducted at +4 °C for 8 h. After immobilization, the electrode surface was rinsed with the HEPES-EDTA buffer solution and pure water (MilliQ) and dried with nitrogen gas. The supramolecular protein assembly consisting of MBP SAM/streptavidin/bio-CRP antigen formed the recognition layer for the subsequent immobilization of anti-CRP antibody.

The target analyte, anti-CRP antibody, was immobilized on the recognition layer by applying a 40 µL drop of anti-CRP antibody in HEPES-EDTA aqueous solution (20 µg/mL) over the electrode area. The immobilization of anti-CRP antibody onto the supramolecular recognition layer was conducted at +4 °C for 8 h. After immobilization, the electrode surface was rinsed with buffer solution and pure water (MilliQ) and dried with nitrogen gas.

### 2.6. Scanning Probe Microscopy

An NTEGRA Prima (NT-MDT, Russia) atomic force microscope (AFM) was used to analyze the topography of the samples in intermittent-contact mode. The images (1,024 × 1,024 pixels) were captured in ambient conditions (RH = 20–26%, T = 24–28 °C) using silicon cantilevers with a nominal tip radius of 10 nm (Model: NSG10, NT-MDT, Russia). The scanning rate and the damping ratio were 0.39 Hz and 0.6–0.7, respectively. The reported values for the root-mean-square roughness (σ, the standard deviation of height features) and the surface area ratio (S_dr_, the increment of the interfacial surface area relative to the area of the projected flat plane) were calculated from 1 µm × 1 µm AFM images. 

### 2.7. X-Ray Photoelectron Spectroscopy

The X-ray photoelectron spectroscopy (XPS) spectra were obtained with a PHI Quantum 2000 scanning spectrometer, using monochromatic Al Kα (1,486.6 eV) excitation and charge neutralization by using an electron filament and an electron gun. The photoelectrons were collected at 45° in relation to the sample surface with a hemispherical analyzer. The analyzing depth was approximately 5–10 nm. The pass energy was 117.4 eV, and the acquisition time was 10 min. The measurements were carried out on three different spots for each sample.

### 2.8. Surface Plasmon Resonance

**Figure 2:**
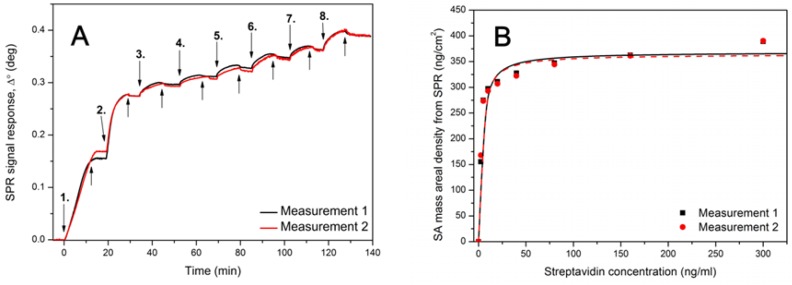
(**A**) Surface plasmon resonance (SPR) signal response after injecting 1.25–300 nM streptavidin in HEPES-EDTA buffer over MuOH:Biotin-PEG-thiol (85:15 mol%) SAM sensor surface. Down arrows represents the time of streptavidin injections: 1. = 2.5 nM, 2. = 5 nM, 3. = 10 nM, 4. = 20 nM, 5. = 40 nM, 6. = 80 nM, 7. = 160 nM and 8. = 300 nM. Up arrows represent the injection of buffer without streptavidin. (**B**) Mass areal density curve for streptavidin showing the Langmuir fit over the data points.

**Figure 3:**
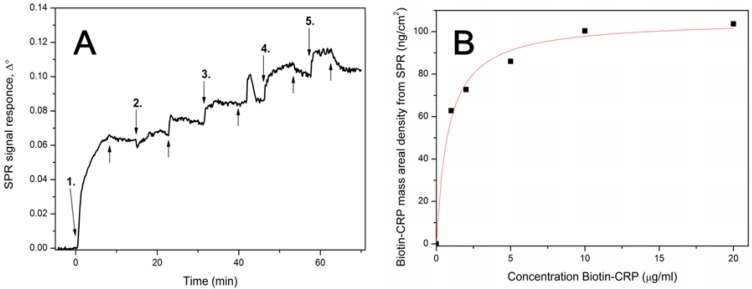
(**A**) SPR signal response after injecting 1–20 μg/mL bio-CRP antigen in HEPES-EDTA buffer over MuOH:Biotin-PEG-thiol (85:15 mol%)/streptavidin sensor surface. Down arrows represents the time of bio-CRP antigen injections: 1. = 1 µg/mL, 2. = 2 µg/mL, 3. = 5 µg/mL, 4. = 10 µg/mL and 5. = 20 µg/mL. Up arrows represents the injection of buffer without bio-CRP antigen. (**B**) Mass areal density of bio-CRP antigen showing the Langmuir fit over the data points.

Surface Plasmon Resonance (SPR) gold slides were coated with SAM by immersing the slide in MBP solution (5 mM in ethanol) for 16 h. After thiolation, the substrates were removed from the solution and rinsed immediately with absolute ethanol and dried with nitrogen gas. Immobilization of streptavidin and bio-CRP-antigen was monitored *in situ* with a SPR instrument (SPR Navi 200, Bionavis Ltd., Tampere, Finland). The SPR Navi 200 instrument has an integrated peristaltic pump and a sample loop system connected to a 6-port valve, which allows injection of sample plugs into the continuously running buffer. For streptavidin and bio-CRP-antigen interaction measurements, the flow channel of the SPR system was first filled with HEPES-EDTA buffer with a constant flow rate of 20 µL/min. After a stable baseline was obtained, solutions with increasing concentrations (ranging from 2.5 to 300 nM for streptavidin, Figure 2, or from 1 to 20 µg/mL for bio-CRP-antigen, Figure 3) were injected as a plug into the continuously flowing buffer stream to measure the specific interaction between streptavidin and the MuOH:Biotin-PEG-thiol (85:15 mol%) SAM sensor surface, or bio-CRP antigen and the MuOH:Biotin-PEG-thiol (85:15 mol%)/streptavidin sensor surface.

### 2.9. Impedance Measurements

Impedance measurements were done on the paper-supported gold electrodes functionalized with a MBP SAM and protein layers in contact with the electrolyte HEPES-EDTA buffer solution. Buffer solution (20 µL) was deposited on the electrode area exactly at the same spot, which had been functionalized with a SAM and the protein layers (Supplementary Figure S1). The real capacitance was measured within a frequency range of 1 Hz to 1 MHz. A Gamry 600 Impedance Spectrometer was used for performing the experiments. An a.c. voltage with an rms amplitude of 20 mV was applied to probe the capacitance and a d.c. bias of 100 mV was applied on top of a.c. voltage.

## 3. Results and Discussion

### 3.1. Binding Capacity of Biofunctional Layers Determined by SPR

The binding capacities of the biofunctional layers included in the supramolecular recognition assembly were determined separately by SPR. This was done to confirm the successful adsorption of proteins and sufficient binding capacity of the individual layers in the recognition system. Figure 2(A) shows an SPR response curve after injecting 1.25–300 nM streptavidin over the MBP thiol SAM surface. Figure 2(B) shows the mass areal density of streptavidin calculated based on SPR response (including the Langmuir adsorption isotherm fit to the data points). The maximum adsorbed amount obtained from the Langmuir fit yielded the value 366 ± 2 ng/cm^2^. This is in the upper range reported by others for streptavidin adsorbed on biotinylated SAMs and solid-supported lipid bilayers, *i.e.*, ~210–370 ng/cm^2^ [23,24,25]. This confirms the favorable orientation and high binding capacity of a MBP thiol SAM towards streptavidin. 

The maximal binding capacity of the immobilized streptavidin layer towards bio-CRP antigen was similarly tested by SPR (Figure 3). The maximum amount of adsorbed bio-CRP antigen obtained from the Langmuir adsorption isotherm fit gave a value of 105 ng/cm^2^. The SPR results show that the bio-CRP antigen adsorbed on the streptavidin surface. The CRP antigen is a 125 kDa doughnut-shape homopentamer composed of five non-covalently associated protomeric subunits arranged around a central pore [26]. The overall crystallographic dimension of the CRP pentamer is about 10.2 nm outside diameter with a protomer diameter of 3.6 nm and a central pore diameter of 3.0 nm [27,28]. The area per molecule expected for a full monolayer of CRP antigen is estimated to be around 125 nm^2^/molecule with preferred planar orientation [29,30]. Using the adsorbed quantity of bio-CRP antigen obtained from the SPR measurements, a value of 186 nm^2^/molecule for the bio-CRP antigen adsorbed on the streptavidin coated MBP thiol SAM was calculated, corresponding to a surface coverage of approximately 67%. 

### 3.2. Topographical and Chemical Characterization of the Immobilized Layers on Paper-Supported Printed Gold Electrodes

**Figure 4:**
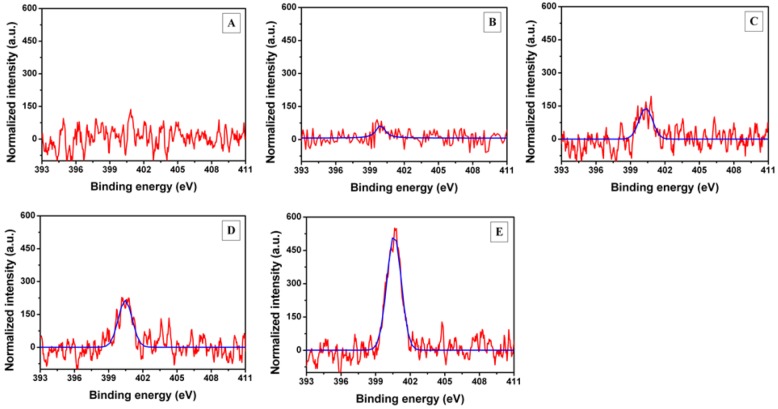
X-ray photoelectron spectroscopy (XPS) N1s peaks for (**A**) gold electrode (**B**) MuOH:Biotin-PEG-thiol (85:15 mol%) SAM, (**C**) streptavidin, (**D**) bio-CRP antigen and (**E**) anti-CRP antibody layers.

Immobilization of the subsequent protein layers on the paper-supported printed gold electrodes was followed by XPS and AFM. Table 1 lists the XPS elemental composition and Figure 4 shows high resolution spectra for the N1s peak after each immobilization step. 

**Table 1:** Atomic percentages of selected elements after each immobilization step.

XPS element	MuOH:Biotin-PEG-thiol (85:15 mol%) SAM	Streptavidin	bio-CRP antigen	anti-CRP antibody
C1s	67.8 ± 4.0%	69.3 ± 3.4%	62.9 ± 3.0%	67.7 ± 4.2%
Au4f	15.6 ± 0.6%	10.2 ± 1.0%	9.0± 0.8%	7.0 ± 1.0%
N1s	0.7 ± 0.4%	2.0 ± 0.3%	4.0 ± 0.8%	7.8 ± 0.5%
S2p	0.3 ± 0.2%	0.2 ± 0.2%	0.1 ± 0.1%	-
O1s	15.8 ± 4.1%	20.3 ± 2.0%	21.2 ± 1.7%	17.5 ± 1.3%

The N1s peak with binding energy of 400 eV can be assigned to the peptide bond (–NH–C(=O)–) of proteins [31,32]. The intensity of the N1s peak increased after each immobilization step, indicating an increase in the amount of proteins on the surface [33]. The magnitude of increase of the N1s peak intensity was approximately the same for streptavidin (85 counts) and bio-CRP antigen layers (90 counts), but over three-fold (300 counts) for anti-CRP antibody (Figure 4). This might reflect the difference in thickness and surface coverage of the adsorbed protein layers, as well as the size difference between different proteins (streptavidin 66 kDa, bio-CRP antigen 125 kDa, anti-CRP antibody 160 kDa). The decrease in the relative amount of the Au4f peak further confirms the supramolecular protein assembly on the gold electrode (Table 1). The presence of the Au4f peak also indicates that the protein layers contained defects induced by the incomplete coverage of the anti-CRP on the streptavidin surface. This leads to occasional protein-free regions on the surface. Such protein-free areas remained even after the immobilization of anti-CRP antibody. The presence of the Au4f peak in the XPS spectrum can also be from the penetration depth of 5–10 nm of the scanning beam, which is comparable to the thickness of the formed layers.

**Figure 5:**
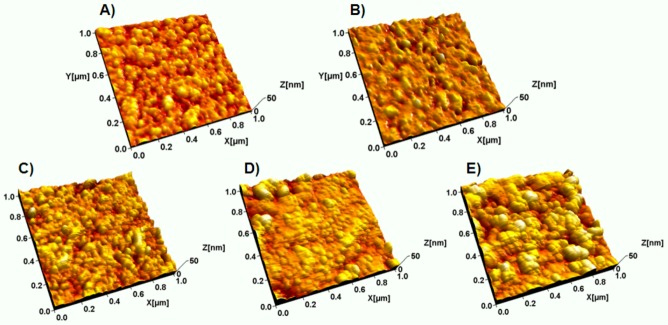
Atomic force microscopy (AFM) topographic images of (**A**) pristine gold electrode, (**B**) MuOH:Biotin-PEG-thiol (85:15 mol%) SAM, (**C**) streptavidin layer, (**D**) bio-CRP antigen layer and (**E**) anti-CRP antibody layer.

Figure 5 shows typical AFM topographs of the electrode surface after each subsequent immobilization step. The roughness values, σ and S_dr_, obtained from the corresponding AFM images are listed in Table 2. A visual observation of the topographs shows that the nano-particulated surface structure of the gold electrode (Figure 5(A)) had been somewhat smoothed after the application of the MBP SAM (Figure 5(B)). However, this did not induce any significant changes in the roughness of the electrode surface (Table 2). 

**Table 2:** Values of selected roughness parameters, including standard deviations, obtained from 1 µm × 1 µm AFM topographs after each immobilization step.

Layer	S_dr_ ± SD [%]	σ ± SD [nm]
gold electrode	1.6 ± 0.2	2.1 ± 0.3
MuOH:Biotin-PEG-thiol (85:15 mol%) SAM	1.4 ± 0.3	2.2 ± 0.5
Streptavidin	3.1 ± 0.5	2.5 ± 0.4
bio-CRP antigen	2.4 ± 0.5	3.3 ± 0.5
Anti-CRP antibody	4.7 ± 0.8	5.4 ± 1.0

**Figure S2:**
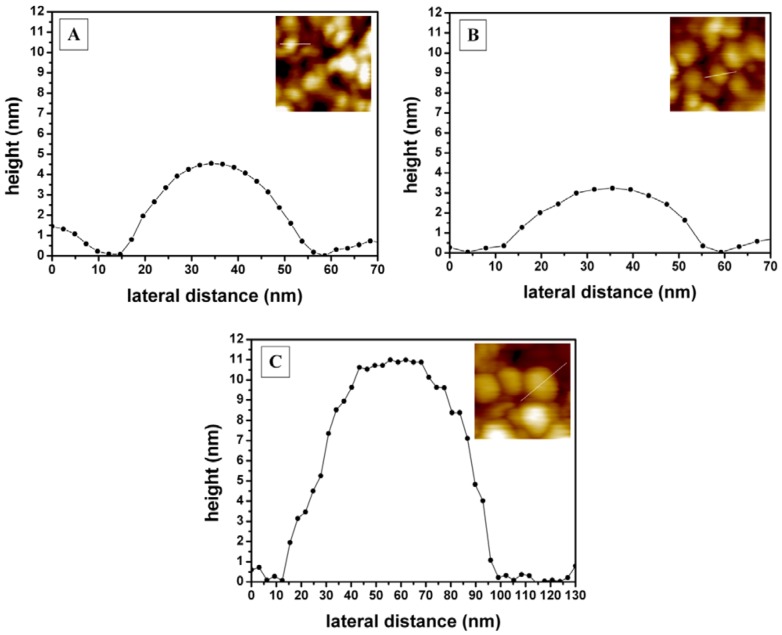
(**A**) A line profile over a single globular object in the AFM topograph after the immobilization of streptavidin. The inset shows the AFM image (200 nm × 200 nm, height scale 6 nm) with a line indicating the location of line profile. (**B**) A line profile over a single globular object in the AFM topograph after the immobilization of bio-CRP antigen. The inset shows the AFM image (200 nm × 200 nm, height scale 8 nm) with a line indicating the location of line profile. (**C**) A line profile over a single globular object in the AFM topograph after the immobilization of CRP antibody. The inset shows the AFM image (200 nm × 200 nm, height scale 19 nm) with a line indicating the location of line profile.

Figure 5(C) shows that after the immobilization of streptavidin, the surface consisted of a quite densely packed layer of globular objects with an average height of 4–6 nm (a typical line profile is depicted in Supplementary Figure S2(A)). The average height value corresponds quite nicely to the crystallographic dimensions of a streptavidin molecule (4.2 nm × 4.2 nm × 5.0 nm) [34]. In addition, the roughness values slightly increased as a result of the adsorption of streptavidin. 

After introduction of the bio-CRP antigen, the surface texture changed and consisted of particles with somewhat larger lateral size variations compared to the streptavidin surface (Figure 5(D)). The change in the surface texture is reflected by an increase in surface roughness (σ) and by a decrease in the effective surface area (S_dr_) (Table 2). The average height of a typical particle on the surface was around 3–4 nm (a typical line profile is depicted in Supplementary Figure S2(B)). The height corresponds well to the height of a CRP antigen previously obtained by AFM and confirms the planar orientation of the adsorbed proteins [29,30]. In addition, larger globular objects (10–13 nm in height) were also observed in AFM topographs. These are either perpendicularly oriented proteins or protein aggregates, indicating a less dense packing. Rather similar height values of the streptavidin and bio-CRP antigen layers are consistent with the increase observed in the corresponding N1s peak intensities (Figure 4).

The immobilization of anti-CRP antibodies increased the presence of larger particles with lower packing density (Figure 5(E)). This is reflected as a clearly increased roughness of the surface (Table 2). A typical y-shaped antibody can be considered to be slightly larger in size (14 nm × 8.4 nm × 4 nm) and mass (160 kDa) compared to CRP antigen [35]. In addition, a single bio-CRP antigen has five similar epitopes that can be recognized by multiple anti-CRP antibodies, leading to an increased average cluster size. The average height of the particles was around 10–14 nm (a typical line profile is depicted in Supplementary Figure S2(C)), indicating a more perpendicular orientation of the anti-CRP antibody molecules that may be caused by a steric hindrance due to adjacent anti-CRP antibody. 

In summary, both XPS and AFM results confirm that a supramolecular assembly was successfully formed on the paper-supported printed gold electrode surfaces, and the bio-CRP antigen layers successfully bound the analyte, *i.e*., the anti-CRP antibody.

### 3.3. Impedimetric Analysis of the Immobilized Layers

The layer coverage of the gold surfaces was electrically probed using impedance spectroscopy measurements. Impedance spectroscopy is a versatile tool for measuring the change in capacitance of multilayered film structures [36]. In the present case, the capacitance was measured in a buffer solution between two gold electrodes on the same substrate (device structure in supplementary information, Figure S1). Real capacitance was measured for a frequency range of 1 Hz to 1 MHz. The change in the capacitance value of the buffer solution was recorded for different gold electrodes. The measurement set-up is the same as used in the previous work [20] where the two metal electrodes were connected through the buffer. Even though capacitive transducing is not as commonly used as amperometric or potentiometric transducing, it has been previously shown to be a very sensitive and effective method for biosensing in various systems [37]. The operating principle of transduction using capacitive measurement is the double-layer formation between the metal and the subsequent layers and the change in capacitance due to that. The variation in the quality of the double layer influences the capacitive values more significantly than the current or voltage variation. We have taken the advantage of the capacitive method previously to clarify the quality of the thiol layers on top of the various Smetal electrodes [20].

**Figure 6:**
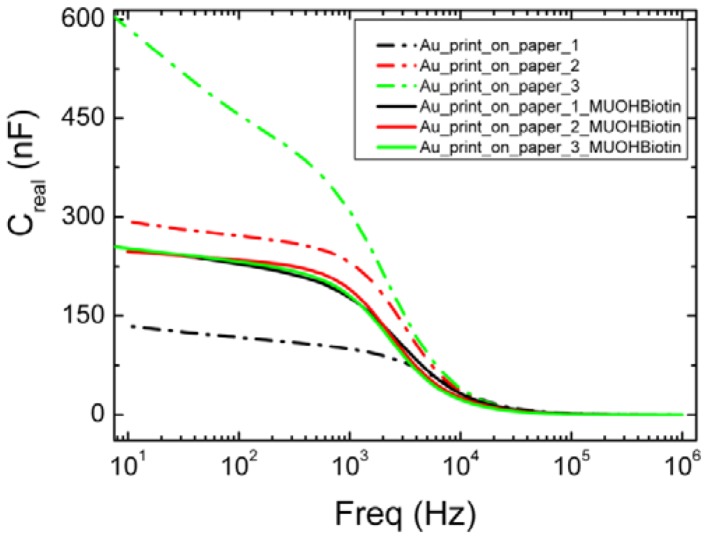
Capacitance as a function of frequency after application of SAM.

The proximity of the aqueous solution to the metal electrode usually indicates a higher capacitance value due to the high polarity of the buffer solution. However, there is a large variation between the capacitive responses of the different batches of paper-supported printed gold electrodes (Figure 6). These electrodes were fabricated using dodecanethiol-capped AuNP ink, and it has been previously shown that after IR sintering, there is a residual thiol layer still present on the surface of the electrodes [19]. The amount varies between the different batches of electrodes. This residual thiol layer is the source for a lower capacitance observed for pristine gold electrodes, as discussed earlier in this work, as well as in previously published work [20]. From Figure 6, we see that there is a dispersion in the capacitance value (increasing capacitance with decreasing frequencies) in the low-frequency region. This can be attributed to the roughness of the electrodes. An interesting observation was made as a consequence of the thiolation of the gold electrodes with MBP thiols. Following thiolation, the gold electrodes were covered by a “dielectric” layer and the capacitance of the structure changed. The quality of the thiol layers governs the change in the capacitance of the device structure. It was observed that all the electrodes, previously exhibiting variations due to a varying amount of residual thiols on the surface, now exhibit perfect dielectric properties with no or very little variation after formation of the thiol-coverage. Moreover, the saturated capacitance values of the thiol-covered electrodes in most cases showed steady capacitance values (~200–230 nF) at lower frequencies, indicating a very good quality of the MBP thiol SAM. 

**Figure 7:**
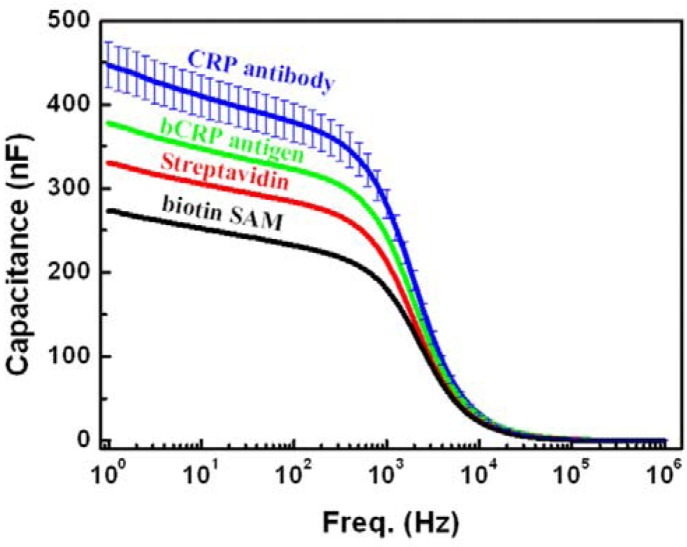
Capacitance as a function of frequency after each immobilized layer.

Following the MBP thiol coverage, the subsequent streptavidin, bio-CRP antigen and anti-CRP antibody layers were consecutively grown on the gold electrodes, as described before. The impedance spectroscopy measurement was done following the formation of each of these layers. Figure 7 shows the systematic variation of the real capacitance for the growth of each successive layer. There is a clear capacitance variation observed in the frequency range of 1 Hz–1kHz. The increase in capacitance cannot be explained using any simple model of equivalent circuit due to the complex nature of the various layers and their interfaces. If we consider capacitance from the double-layers from each interface to add up to a simple capacitive series, then a simple calculation shows that the capacitance for each consecutive layer should decrease the capacitance, not increase it, as seen in Figure 7. The complex nature of the structure would require detailed analysis of the equivalent circuit, which is beyond the scope of the article. The important conclusion is that the impedance measurements show a systematic variation of real capacitance for each consecutive layer growth. This enables the use of the electrical (impedimetric) method for detecting analytes with hand-held, point-of-care diagnostic devices. 

## 4. Conclusions

We have successfully demonstrated the formation of supramolecular protein layers on inkjet printed gold electrodes on paper substrates. We have additionally detected the formation of consecutive layers using an electrical (impedimetric) technique. The formation of the consecutive layers has also been verified and supplemented by SPR measurements and surface sensitive chemical and topographical measurements. Even though, the exact composition and scheme of the layers might not remain the same, but the possibility of building up and detecting supramolecular protein layers on paper and using impedimetry is demonstrated through this article. This provides a possible route for fabricating inexpensive, recyclable, point-of-care diagnostic devices.
